# Comparison of Giemsa microscopy with nested PCR for the diagnosis of malaria in North Gondar, north-west Ethiopia

**DOI:** 10.1186/1475-2875-13-174

**Published:** 2014-05-07

**Authors:** Abebe Alemu, Hans-Peter Fuehrer, Gebeyaw Getnet, Afework Kassu, Sisay Getie, Harald Noedl

**Affiliations:** 1 Department of Medical Parasitology, School of Biomedical and Laboratory Sciences, College of Medicine and Health Sciences, University of Gondar, Gondar, Ethiopia; 2 Institute of Parasitology, Department of Pathobiology, University of Veterinary Medicine Vienna, Vienna, Austria; 3 Department of Medical Microbiology, School of Biomedical and Laboratory Sciences, College of Medicine and Health Sciences, University of Gondar, Gondar, Ethiopia; 4 Institute of Specific Prophylaxis and Tropical Medicine, Medical University of Vienna, Vienna, Austria

## Abstract

**Background:**

Malaria remains one of the leading communicable diseases in Ethiopia. Early diagnosis combined with prompt treatment is one of the main strategies for malaria prevention and control. Despite its limitation, Giemsa microscopy is still considered to be the gold standard for malaria diagnosis. This study aimed to compare the performance of Giemsa microscopy with nested polymerase chain reaction (nPCR) for the diagnosis of malaria in north-west Ethiopia.

**Methods:**

A cross sectional study was conducted in public health facilities in North Gondar, from March 2013 to April 2013. A total number of 297 subjects with suspected malaria were enrolled in the study. Finger-prick blood samples were collected and examined for *Plasmodium* parasites using Giemsa microscopy and standard nPCR.

**Results:**

Among the study participants, 61.6% (183/297) patients tested positive for malaria by Giemsa microscopy of which, 72.1% (132/183) and 27.9% (51/183) were diagnosed as *Plasmodium falciparum* and *Plasmodium vivax*, respectively. By nPCR, 73.1% (217/297) were malaria-positive. Among microscopy-negative samples, 13.1% (39/297) samples turned malaria-positive in nPCR. In nPCR, the rate of mixed *Plasmodium* infections was 4.7% (14/297) and 3.03% (9/297) were positive for *Plasmodium ovale*. Using nPCR as reference the sensitivity, specificity, positive predictive and negative predictive values of Giemsa microscopy were 82.0%, 93.8%, 97.3% and 65.8%, respectively, with a good agreement (κ = 0.668) to nested PCR. The sensitivity and specificity of Giemsa microscopy in identifying

*P. falciparium* infections were 74.0% and 87.4% and 63.2% and 96.5% for *P. vivax* infections, respectively.

**Conclusion:**

Although Giemsa microscopy remains the gold standard for malaria diagnosis in resource-limited environments, its sensitivity and specificity as compared to nPCR is limited suggesting exploration of novel rapid and simplified molecular techniques for malaria-endemic countries. A high rate of misclassification and misidentification highlights the importance of adequate training for staff involved in malaria diagnosis.

## Background

Malaria is a disease caused by protozoans of the genus *Plasmodium* and continues to be one of the main causes of serious illness and death throughout the world. In spite of considerable improvements in some parts of the world, malaria-related morbidity and mortality have increased in other regions [1]. Malaria varies widely in epidemiology and clinical manifestations in different parts of the world. This variability is mainly due to differences in the species and prevalence of malaria parasites in different localities, difference in the susceptibility of *Plasmodium* to anti-malarial drugs, the distribution of mosquito vectors and immune status of the population [2].

Malaria is also among the leading communicable diseases in Ethiopia. It is estimated that 57.3 million (68%) of the population of Ethiopia live in areas at risk of malaria [3]. The Federal Ministry of Health (FMOH) estimates that there are 5–10 million clinical malaria cases each year [4] accounting for 12% of outpatient consultations and 10% of admissions to hospitals [5]. In Ethiopia, *Plasmodium falciparum* and *Plasmodium vivax* are the main species accounting for approximately 60% and 40% of malaria cases, respectively [6] but changing epidemiology has resulted in a major shift from *P. falciparum* to *P. vivax* in the country [7]. Nonetheless, *P. falciparum* is the main causative of severe malaria in Ethiopia with case fatality rates of about 10% in hospitalized adults and up to 33% in children less than 12 years old [8].

Early and adequate diagnosis and prompt treatment is one of the main strategies in malaria prevention, control and effective disease management. In Ethiopia, different diagnostic methods are employed but diagnosis based on clinical signs and symptoms is still most frequently used in all peripheral areas where laboratory diagnosis is not available. However, clinical diagnosis is notoriously unreliable as the sign and symptoms of malaria are non-specific and overlapping with those of other febrile diseases. In recent years the use of rapid diagnostic tests (RDTs) has gained major importance wherever laboratory diagnosis is not available (e.g. at health posts). However, microscopic diagnosis of malaria based on examination of Giemsa-stained blood films remains the gold standard in Ethiopia [9].

The sensitivity of Giemsa microscopic slide examination significantly varies across species and geographic localities. A study in Ethiopia reported a sensitivity that varied between 44% and 96%, and a specificity of greater than 90% [10]. Microscopy offers significant advantages to other methods (like RDTs), but it has its own limitations, including: detection of low parasite loads, result interpretation, mixed infections and limited usefulness in non-endemic regions due to frequently inadequate training and experience of laboratory personnel [11].

An earlier study from north-west Ethiopia suggested that malaria microscopy leads to 16.3% false negative, 0.7% of false positive results and the misclassification of *Plasmodium* species [12]. The aim of this study was, therefore, to compare Giemsa microscopy to nested polymerase chain reactions (nPCR) for the diagnosis of malaria in North Gondar, north-west Ethiopia, to provide a better understanding of the reliability of Giemsa microscopy for malaria diagnosis in this region.

## Methods

### Study area

The study was conducted in a hospital (Metema hospital) and three health centres (Maksegnt, Enfranze and Kola Diba) in North Gondar areas which are known to be endemic for malaria. The altitude ranges from 1,750 to 2100 m above sea level. According to the Municipal Health Bureau report, malaria is the most prevalent seasonal disease in these areas.

### Study design, inclusion and exclusion criteria

A diagnostic study was conducted from March 2013 to April 2013. Male and female febrile patients of any age suspected to be positive for malaria, willing to participate and to sign the informed consent were included in this study. Patients who had received anti-malarial drugs during the past four weeks and critically ill patients unable or unwilling to provide a blood sample were excluded from the study.

### Sample size and sampling techniques

Study participants were recruited consecutively (convenience sampling) and a total of 297 patients with signs and symptoms consistent with malaria enrolled. Finger-prick blood samples were collected from every participant and placed on grease-free, clean microscopic glass slides. On a single slide, both thick and thin blood films were prepared. The thin blood films were fixed in methanol after air-drying before the slides were stained in a 30% Giemsa solution for 10 minutes. Thin and thick blood films were read at the health centre by experienced medical laboratory technologists and the result was considered as negative if no *Plasmodium* parasites were seen after examining 100 high power (1000x) fields.

### Sample collection for molecular analysis

Two blood spots were collected from each participant, transferred on filter paper (Whatman #903, GE Healthcare) labelled with the participant’s study code and date. Each filter paper was dried individually to avoid any chance of contamination. The samples were then stored in small plastic bags with desiccant and transported to the Institute of Specific Prophylaxis and Tropical Medicine, Medical University of Vienna (MUV), Vienna, Austria for molecular analysis.

### DNA isolation and parasite detection by nested PCR

A modified chelex-based DNA extraction method using the InstaGene Whole Blood Kit (Bio-Rad Laboratories, Hercules, CA, USA) was used for the extraction and purification of *Plasmodium* DNA from the blood spots on filter paper. Parasite detection and species classification by nested PCR assay was performed for all samples as described previously [13,14]. The individual interpreting the PCR results was blinded to the results of microscopy.

### Data analysis

Sensitivity, specificity and predictive values were determined using SISA online statistical software [15]. The kappa coefficient (Cohen's kappa coefficient as a measure of agreement for qualitative items) was determined to confirm the consistency of the results among the diagnostic tools.

### Ethical consideration

The study protocol had been reviewed and approved by the institutional review board (IRB) of the University of Gondar prior to the study. Written informed consent (consent and/or assent) was obtained from all participants or their legal guardians after being translated and read in the vernacular language. Patients testing positive for malaria by microscopy received immediate treatment according to the national treatment guidelines.

## Results

### Comparison of microscopy and nested polymerase chain reaction for the diagnosis of malaria and *Plasmodium* species identification in Northwest Ethiopia

**Figure 1:**
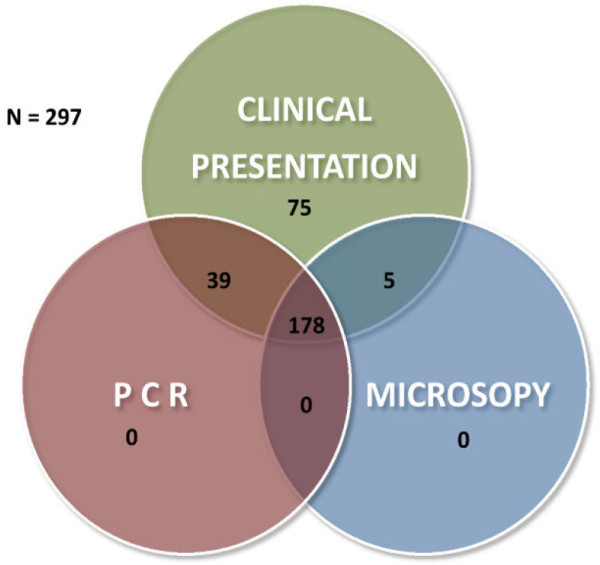
Venn diagram showing relationship between positivity in clinical presentation, Giemsa microscopy and nPCR.

A total of 297 febrile patients with suspected malaria based on clinical presentation were enrolled and screened for *Plasmodium* parasites in the course of the study. Among the study participants, 61.6% (183/297) patients were malaria-positive by microscopy. Of those, 72.1% (132/297) and 27.9% (51/297) were diagnosed as *P. falciparum* and *P. vivax*, respectively, by Giemsa microscopy. Nested PCR was used as reference method and 73.1% (217/297) participants tested positive by nested PCR. Among microscopy-negative patients, 13.1% (39/297) cases tested positive by nested PCR (Figure 1). In this study, Giemsa microscopy did not identify any mixed infections of *Plasmodium* parasites or any cases of *Plasmodium ovale* infections. However, 4.7% (14/297) samples were identified as mixed *Plasmodium* species infections by nested PCR and *P. ovale* was in 3.03% (9/297) patient samples (Table 1).

**Table 1:** Comparison of microscopy and nested polymerase chain reaction for malaria parasite detection and species identification in Northwest Ethiopia, 2013

**Parasites detected by microscopy and nested PCR (Number of samples)**	
Microscopy (n)	Nested PCR (n)
*Plasmodium falciparum* (132)	*P. falciparum* (108), *P. vivax* (11), *P. ovale* sp. (2), *P. falciparum* + *P. vivax* (6), negative (5)
*Plasmodium vivax* (51)	*P. vivax* (38), *P. falciparum* (4), *P. ovale* sp. (2), *P. vivax* + *P. falciparum* (5), *P. falciparum* + *P. ovale* sp. (2)
Negative (114)	Negative (75), *P. falciparum* (28), *P. vivax* (8), *P. ovale* sp. (2), *P. falciparum* + *P. ovale* sp. (1)

n = number of blood samples examined.

### Agreement, sensitivity, specificity and predictive values of Giemsa microscopy compared to nested PCR for *Plasmodium* detection and *Plasmodium* species identification

Giemsa microscopy showed a good measure of agreement (κ = 0.668) with the reference method, nested PCR for *Plasmodium* detection. Its sensitivity and specificity were 82.0% (95% CI: 76.1-86.8) and 93.8% (95% CI: 85.4-97.7), respectively. The probability for a positive microscopy result to be true positive (positive predictive value, PPV) was 97.3% (95% CI: 93.4-99.0) and for a negative result to be true negative (negative predictive value, NPV) was 65.8% (95% CI: 56.2-74.3.1).

Taking mixed infections into account (a mixed infection counting for both species as positive) the *P. falciparum*-specific sensitivity of microscopy relative to nPCR was 74.0% (95% CI: 66.2-80.6%), the specificity 87.4% (95% CI: 80.6-92.2%), the positive and negative predictive values 86.4 (95% CI: 79.0-91.5%) and 75.8 (68.4-81.9%), respectively. The corresponding values for *P. vivax* were 63.2% 95% CI: (50.6-74.4%) for sensitivity, 96.5% (95% CI: 93.0-98.4%) for specificity, 84.3% (95% CI: 70.9-92.5%) for PPV, and 89.8% (95% CI: 85.2-93.2%) for NPV.

## Discussion

Malaria is still a leading cause of morbidity in Ethiopia where 78% of the country and 68% of its population are at risk of malaria. *Plasmodium falciparum* contributes around 60-75% to the malaria burden and the only other species routinely identified being *P. vivax*[16,17]. As diagnostic resources are limited throughout Ethiopia, microscopy remains the laboratory standard for diagnosing malaria [18]. However, even under optimal conditions the sensitivity of microscopy is limited to approximately 20 parasites/μl of blood, and subjective interpretation and reader errors further reduce the accuracy of diagnosis [19]. Nested PCR has a somewhat lower limit of detection of approximately 6 parasites/μl of blood when using dried blood sample [20,21] which depends on the sample used (e.g. dried *versus* fresh). In the current study, whole blood collected on filter paper has been used for nested PCR. The difference in sensitivity is obviously reflected in the different rates of positivity seen in this study (61.6% by microscopy *versus* 73.1% in nPCR).

Nested PCR utilizes genus and species specific markers for the detection of *Plasmodium* parasites. This allows for the detection of low density infections and even more importantly of mixed infections, which are routinely missed in microscopy [13] and makes nested PCR an ideal confirmatory test for malaria diagnosis. In the present study, 39 (13.1%) sample considering negative by microscopy gave positive results in nested PCR.

These results are in concordance with those of other studies from Africa, Asia and Latin America reporting a considerably higher potential for detecting low parasite densities [22-26]. A recent study from Ethiopia suggests that PCR has a much higher potential for detecting sub-clinical infections [27].

In the present study, the sensitivity of Giemsa microscopy for the diagnosis of *P. falciparum* was considerably higher when compared to *P. vivax* (74.0% vs. 63.2%), while at the same time the specificity was higher for *P. vivax* (87.4% vs. 96.5%) suggesting that microscopists tend to interpret slides that are difficult to read (either because of morphology, artefacts, or low parasitaemia) as *P. falciparum* rather than *P. vivax.*

Although Giemsa microscopy did not identify a single case of mixed infection, all *P. vivax-P. falciparum* infections were read as malaria-positive and approximately half of them were interpreted as *P. falciparum* monoinfections, the other half as *P. vivax*. This is somewhat surprising as previous experience shows that mixed infections tend to be interpreted as *P. vivax* rather than *P. falciparum*, obviously because of its characteristic morphology.

Until very recently, species other than *P. falciparum* and *P. vivax* had not been reported from the region [12]. This is in part due to the fact that training of microscopists largely focuses on the detection of the two most prevalent species but also on the fact that molecular data to confirm and/or augment microscopic results are rarely available. In this study, nine samples tested positive for *P. ovale* out of which six were monoinfections and three were mixed with *P. falciparum*. However, only three were interpreted as being negative by microscopy. Once again not a single sample tested positive for *Plasmodium malariae*.

In spite of the inherent limitations of malaria Giemsa microscopy, the quality of microscopic diagnosis largely depends on the quality of training [28]. On the one hand, adequate training can increase the yield of accurate malaria diagnosis which helps to reduce illness, potential death, mistreatment and persistently high disease burden while at the same time saving vital resources for malaria control [29,30].

The added cost of using molecular techniques as confirmatory diagnostic tool for the malaria control programs needs to be carefully balanced against the cost (not to mention the human cost) of a higher disease burden. In the near future PCR (or equivalently sensitive diagnostic tools) will not be available throughout most malaria-endemic regions but a network of reference centers could potentially support ongoing diagnostic and control efforts by malaria control programs on the long run even significantly reduce the overall cost by providing a more targeted approach to the challenges of the malaria eradication era.

## Competing interests

The authors declare that they have no competing interests.

## Authors’ contributions

AA and HN conceived the study. AA and HFP undertook statistical analysis, undertook molecular analysis of the samples and drafted the manuscript. GG, SG and AK contributed in proposal writing, blood sample collection and microscopic diagnosis of malaria slides. All authors contributed to the writing of the manuscript and approved the submitted version of the manuscript.
